# Evaluation of Serum AMH, INHB Combined with Basic FSH on Ovarian Reserve Function after Laparoscopic Ovarian Endometriosis Cystectomy

**DOI:** 10.3389/fsurg.2022.906020

**Published:** 2022-05-18

**Authors:** Yan Tang, Yanning Li

**Affiliations:** Department of Obstetrics and Gynecology, The Third Affiliated Hospital of Chongqing Medical University, Chongqing, China

**Keywords:** ovarian endometriotic cyst, anti-mullerian hormone, inhibin B, follicle stimulating hormone, basic FSH

## Abstract

**Objective:**

The value of serum AMH, INHB, and bFSH levels in assessing postoperative ovarian reserve function was analyzed by measuring serum anti-Mullerian hormone (AMH), inhibin B (INHB), and basal follicle-stimulating hormone (bFSH) levels in patients after laparoscopic cystectomy for endometrioma.

**Methods:**

From June 2019 to December 2021, 124 patients underwent laparoscopic cystectomy for endometrioma in our hospital were selected, and the serum AMH, INHB, bFSH level, antral follicle count (AFC) of all patients before and after operation were detected and compared. According to the results of postoperative testing, all the patients were divided into normal group (*n* = 86), diminished ovarian reserve (DOR) group (*n* = 27), and premature ovarian failure (POF) group (*n* = 11). Pearson correlation model and subject operating characteristic curve (ROC) were used to analyze the correlation and diagnostic value of serum AMH, INHB and bFSH levels with postoperative ovarian reserve function, respectively.

**Results:**

After operation, the levels of serum AMH, INHB and AFC in the DOR group and POF group decreased compared with those before the operation, and the serum bFSH levels increased (*p *< 0.05). After operation, the levels of serum AMH, INHB and AFC in DOR group and POF group were lower than those in normal group,and the serum bFSH levels were higher than the normal group; the levels of serum AMH, INHB and AFC in POF group were lower than those in DOR group, and the serum bFSH levels were higher than the DOR group (*p *< 0.05). Pearson analysis showed that serum AMH and INHB levels were negatively correlated with bFSH, and positively correlated with the number of AFC, the serum bFSH level was negatively correlated with the number of AFC (*p *< 0.05). The diagnostic values of serum AMH, bFSH, INHB and the combination of the three tests for postoperative abnormal ovarian reserve function were 0.866 (95% CI, 0.801–0.923), 0.810 (95% CI, 0.730–0.890), 0.774 (95% CI, 0.687–0.860) and 0.940 (95% CI, 0.900–0.981), respectively.

**Conclusion:**

Serum AMH and INHB levels decreased and bFSH levels increased in patients after laparoscopic cystectomy for endometrioma, both of which were closely related to postoperative ovarian reserve function, and both could evaluate ovarian reserve function after ovarian cyst debulking, and the combined test could significantly improve the detection rate.

## Introduction

Ovarian endometriotic cysts (OEMC), or chocolate cysts, are the most predominant pathological type of endometriosis, and OEMC mainly presents with dysmenorrhea, non-menstrual chronic pelvic pain, and in severe cases, infertility, which seriously affects the physical and mental health of patients (1). Laparoscopic debridement of cysts is currently the best method for the treatment of ovarian endometrial cysts, with the advantages of superior results, low postoperative recurrence rate and minimal trauma. Although surgery can remove lesions and relieve symptoms, postoperative risk events such as formation of venous thrombosis, intraoperative bleeding, postoperative infection, adhesion formation, and postoperative reduction of ovarian reserve function and recurrence of postoperative endometriosis can occur (2, 3). Reduced ovarian reserve function is a key factor in infertility in patients with endometriosis; therefore, it is important to fully grasp the postoperative ovarian reserve function of patients and to provide targeted treatment.

Anti-mullerian hormone (AMH) is considered to be an ideal indicator to evaluate ovarian reserve function and is positively correlated with the number of small antral follicles, which can reflect the number of early follicles in the ovary and the survival of primordial follicles (4, 5). Inhibin B (INHB) is produced by small antral follicles, in addition to assessing ovarian reserve, it can also stabilize T cell function and inhibit the overactivation of immune factors from causing damage to ovarian epithelial tissue (6, 7). Basic follicle stimulating hormone (bFSH) promotes follicle maturation, regulates growth and development, and is likewise used to assess ovarian reserve function (8). In this study, we investigated the value of serum AMH, INHB, and bFSH levels in assessing the postoperative ovarian reserve function of patients after laparoscopic ovarian endometriosis cyst debulking by measuring their serum AMH, INHB, and bFSH levels. The specific research is shown as follows.

## Materials and Methods

### General Information

124 patients who underwent laparoscopic ovarian endometrioma cystectomy in our hospital from June 2017 to December 2019 were selected, aged 21–39 years, with an average age of (28.17 ± 5.71) years. Inclusion criteria: a diagnosis of OEMC or other benign ovarian lesions based on the patient's clinical presentation and imaging.; unilateral cystic lesions; normal menstruation; no history of taking sex hormone drugs within 3 months; no history of uterine-related surgery. Exclusion criteria: combined with endocrine diseases; combined with ovarian malignant tumors and other malignant tumors; patients with abnormal ovarian function such as polycystic ovaries; history of hormonal drug use within 6 months and history of pregnancy and lactation.

### Research Methods

Cyst removal: All patients underwent laparoscopic cyst removal by the same group of physicians, i.e., the patient was put under general anesthesia, a pneumoperitoneum was established, a laparoscope was placed, the ovarian cortex was opened with an electric needle after exploration. The ovarian cortex was cut, the cyst wall was removed, the wound was electrocoagulated to stop bleeding, and the cyst was removed.

Serum AMH, INHB, bFSH levels and small antral follicle count (AFC) detection: 5 mL of cubital venous blood was drawn from the patient on the 2nd or 3rd day of preoperative menstruation and on the 2nd or 3rd day of postoperative menstrual period, and then centrifuged for 20 min at 3,000 r/min after 2 h resting. Serum AMH and INHB levels were measured by ELISA (kits purchased from Shanghai Kanglang Biotechnology Co., Ltd.), serum bFSH levels were measured by radioimmunoassay (kits purchased from Shanghai mlbio Co., Ltd.), and AFC was examined by ultrasound. According to the postoperative detection results, they were divided into normal group (86 cases), diminished ovarian reserve (DOR) group (27 cases), and premature ovarian failure (POF) group (11 cases). According to our standard determination, the normal levels of AMH were 2.00–10.00 ng/mL, INHB were 20–175 pg/mL, bFSH were 4–12 U/L, and AFC were ≥4. Normal group: all indexes and menstruation were normal. dOR group: serum bFSH was 12–40 U/L, AFC <4, regular menstrual cycle; POF group: serum bFSH >40 U/L, amenorrhea >6 months.

### Statistical Methods

SPSS 22.0 software was used for processing data. The measurement data were expressed as, and the comparison was performed by one-way ANOVA, and the pairwise comparison was performed by the SNK-q method. Enumeration data were expressed as frequency and percentage [*n*(%)], and the *χ*^2^ test was used. Correlation analysis was performed by using Pearson correlation analysis. The receiver operating characteristic curve (ROC) was drawn, and the area under the ROC curve (AUC) was used to evaluate the diagnostic value of serum AMH, INHB, and bFSH levels on ovarian reserve, and AUC > 0.7 was considered diagnostic significance. *p* < 0.05 means the difference was statistically significant.

## Results

### Comparison of Postoperative Serum AMH, INHB, bFSH, and AFC in Three Groups of Patients

**Figure 1:**
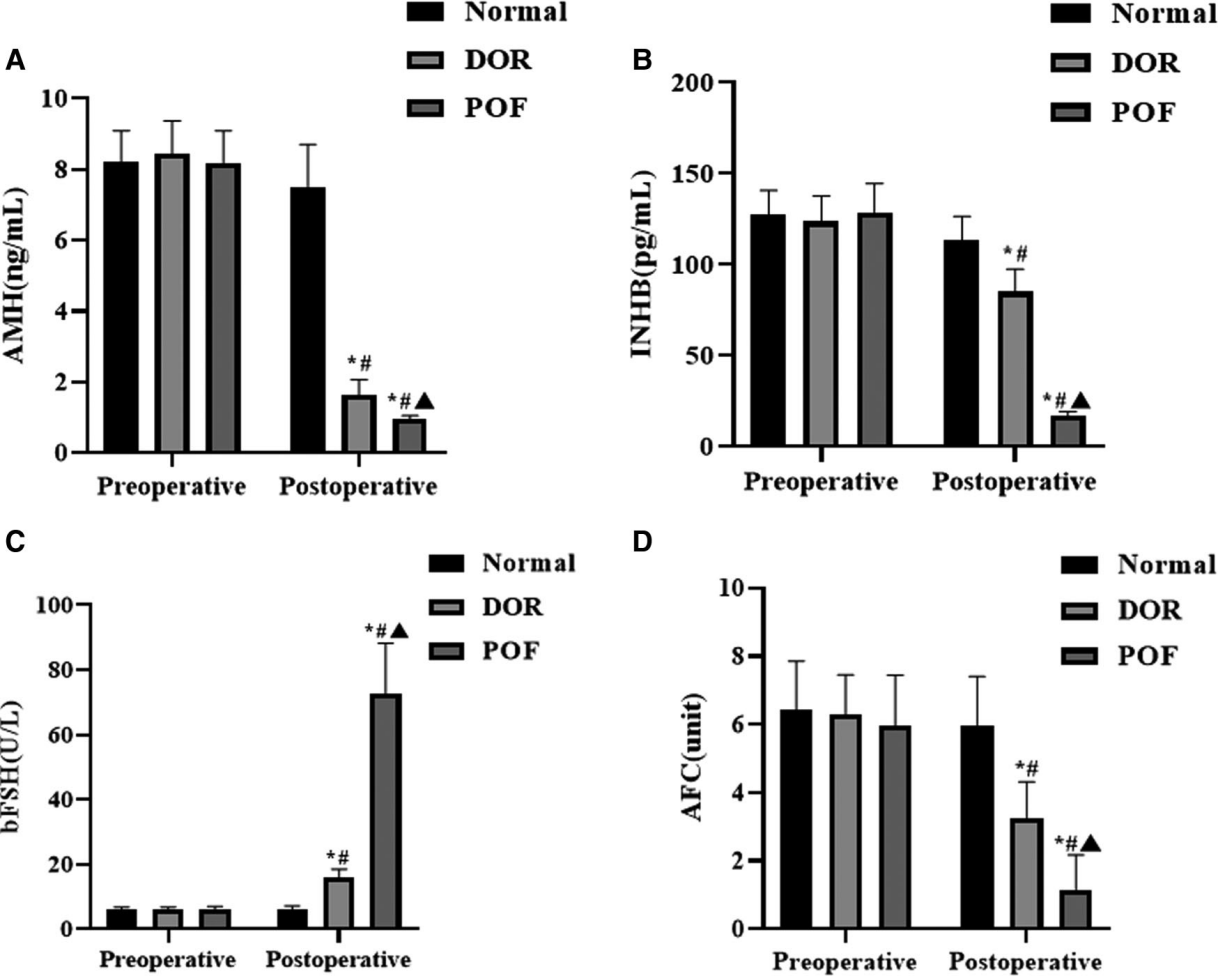
Comparison of postoperative serum AMH, INHB, bFSH and AFC in patients (*n*, $\bar{x}\pm s$). Note: (**A**) is AMH, (**B**) is INHB, (**C**) is bFSH, and (**D**) is AFC. Compared with the same group preoperatively *p < 0.05; compared with normal group after operation, #*p* < 0.05; compared with DOR group after operation, ^▴^*p *< 0.05.

Preoperatively, there were no significant differences in serum AMH, INHB, bFSH levels and AFC counts among the three groups (*p* > 0.05). Postoperative serum AMH, INHB levels and number of AFC in the DOR and POF groups decreased compared with those before surgery and were lower than those in the normal group in the same period, while serum bFSH levels increased and were higher than those in the normal group in the same period, and the differences were statistically significant (*p* < 0.05). The postoperative serum AMH, INHB levels and number of AFC in the POF group were lower than those in the DOR group, and the serum bFSH levels were higher than those in the DOR group, and the differences were statistically significant (*p* < 0.05). See Figure 1.

### Correlation Analysis Between Serum AMH, INHB, bFSH Levels and Postoperative Ovarian Reserve

Pearson analysis showed that serum AMH and INHB levels were negatively correlated with bFSH, and positively correlated with the number of AFCs; serum bFSH level was negatively correlated with the number of AFCs, as shown in Table 1.

**Table 1:** Correlation analysis between serum AMH, INHB, bFSH levels and postoperative ovarian reserve.

Indicator	AMH		INHB		bFSH	
	*r*	*p*	*r*	*p*	*r*	*p*
bFSH	−0.615	0.011	−0.742	0.002	1	<0.001
AFC	0.659	0.007	0.718	0.003	−0.597	0.018

### The Diagnostic Value of Serum AMH, INHB Combined With bFSH on Postoperative Ovarian Reserve

**Figure 2:**
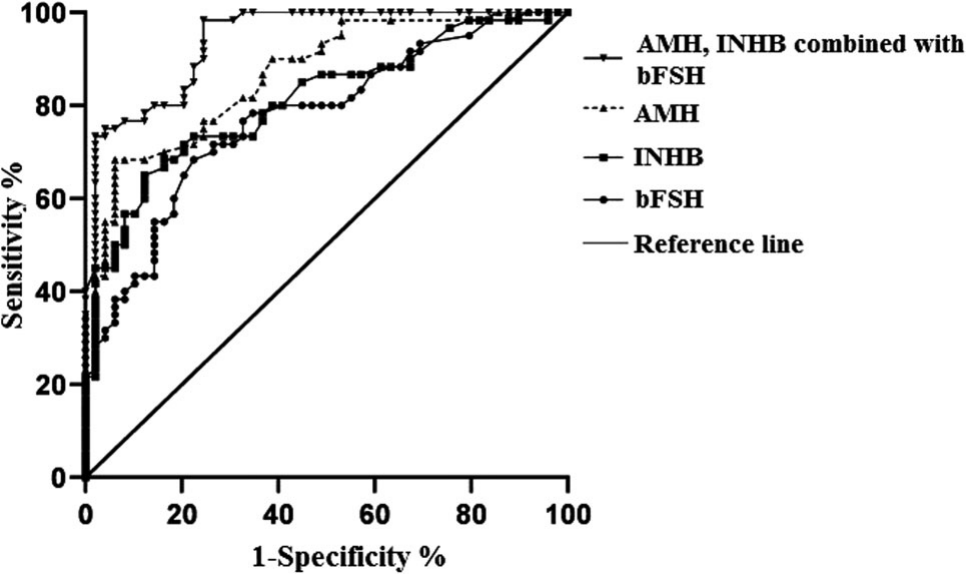
The diagnostic value of serum AMH, INHB combined with bFSH on postoperative ovarian reserve.

The AUC of the predictive value of serum AMH for postoperative abnormal ovarian reserve function was 0.866 (95% CI, 0.801–0.923), with a sensitivity of 88.10% and specificity of 88.30% when the best cut-off value was 0.621; the AUC of the predictive value of serum INHB for postoperative abnormal ovarian reserve function was 0.810 (95% CI, 0.730–0.890), with a sensitivity of 65.00% and specificity of 87.80% when the best cut-off value was 0.528; the AUC of the predictive value of serum bFSH for abnormal postoperative ovarian reserve function was 0.774 (95% CI, 0.687–0.860), with a sensitivity of 68.30% and specificity of 77.60%. The AUC of the predictive value of serum AMH and INHB combined with bFSH for abnormal postoperative ovarian reserve function was 0.940 (95% CI, 0.900–0.981), and when the best cut-off value was 0.638, the sensitivity was 90.10% and the specificity was 89.20%. See Table 2 and Figure 2.

**Table 2:** Diagnostic value of serum AMH, INHB combined with bFSH for postoperative ovarian reserve.

Indicator	AUC	Asymptotic 95% confidence interval		Best cutoff	Sensitivity (%)	Specificity (%)
		Lower limit	Upper limit			
AMH	0.866	0.801	0.932	0.621	88.10	88.30
INHB	0.810	0.730	0.890	0.528	65.00	87.80
bFSH	0.774	0.687	0.860	0.459	68.30	77.60
Serum AMH, INHB combined with bFSH	0.940	0.900	0.981	0.638	90.10	89.20

## Discussion

Laparoscopic treatment has a high clearance rate for OEMC, but because of the tight connection between the cyst and normal ovarian tissue, intraoperative operations such as cyst wall stripping and hemostasis can easily cause follicle loss, resulting in compromised ovarian reserve function in patients (9, 10). Therefore, strengthening the screening of ovarian reserve after cyst dissection is of great significance for early intervention and improvement of patients’ prognosis.

AMH is secreted by granulosa cells and its levels are relatively stable during the menstrual cycle. Its level reflects the number of primary follicles and regulates follicle maturation and development, thus it can be used as a serological marker to assess ovarian reserve function and ovarian responsiveness during ovulation (11, 12). INHB is also secreted by granulosa cells and its abnormal level is closely related to the abnormal function of ovarian granulosa cells, which can feedback inhibit the production of bFSH, while bFSH can promote the production of INHB by granulosa cells. In addition, INHB can also regulate the secretion of estradiol (E2), which can be used to evaluate ovarian reserve function (13, 14). AFC is a proven reliable index to assess ovarian reserve function, negatively correlated with patient age and closely related to ovarian responsiveness during the ovulatory cycle, which can truly reflect the patient's ovarian functional reserve (15). The results of this study showed that postoperative serum AMH, INHB levels and number of AFC in the DOR and POF groups decreased compared with those before surgery and were lower than those in the normal group in the same period, while serum bFSH levels increased and were higher than those in the normal group in the same period. The postoperative serum AMH, INHB levels and number of AFC in the POF group were lower than those in the DOR group, and the serum bFSH levels were higher than those in the DOR group Pearson analysis showed that serum AMH and INHB levels were negatively correlated with bFSH, positively correlated with AFC count, and serum bFSH level was negatively correlated with AFC count. All of these results indicate that serum AMH, INHB, and bFSH are all associated with ovarian function after laparoscopic ovarian endometriosis cyst debulking and that their serological levels change with changes in postoperative ovarian reserve function. It has been suggested that reduced serum AMH and INHB levels are associated with abnormal function of follicular granulosa cells, while reduced serum INHB levels make them less inhibitory to serum bFSH, which in turn causes abnormal ovarian reserve function in patients (16, 17).

Serum bFSH can be used as one of the important indicators for evaluating ovarian reserve function, but some studies (18) found that the serum bFSH level of some patients did not increase after cystectomy. Analysis of the reasons for this may be related to the fact that the patient had only one ovary removed and the other ovary could replace the function of the affected ovary. Therefore, the evaluation of ovarian reserve function after ovarian cyst debulking using serum bFSH alone has the problem of poor specificity and sensitivity. Both Serum AMH and INHB belong to the transforming growth factor-β superfamily, and both serum levels are positively correlated with ovarian responsiveness and follicle number, both of which can be used to assess ovarian reserve function (19, 20). The results of this study found that the AUC of serum AMH, INHB, bFSH and the combination of the three for abnormal ovarian reserve after ovarian cystectomy were 0.866, 0.810, 0.774, and 0.940, respectively, which indicated that compared with serum bFSH, INHB and AMH alone for predicting ovarian reserve function after ovarian cyst debulking, the combined test significantly improve the detection rate of abnormal postoperative ovarian reserve function.

In conclusion, serum AMH and INHB levels decreased and bFSH levels increased in patients after laparoscopic cystectomy for endometrioma, which were closely related to postoperative ovarian reserve function and could be evaluated for ovarian reserve function after ovarian cystectomy, and the combined test can significantly improve the detection rate and facilitate the targeted treatment of patients.

## Data Availability

The original contributions presented in the study are included in the article/Supplementary Material, further inquiries can be directed to the corresponding author/s.
